# Pembrolizumab-induced Stevens-Johnson syndrome-like reaction: An atypical clinical presentation

**DOI:** 10.1016/j.jdcr.2024.10.004

**Published:** 2024-10-28

**Authors:** Arjun Mahajan, Ryan Chen, Grant M. Fischer, Yuqing Xiong, Sepideh Ashrafzadeh, Jordan T. Said, Vinod E. Nambudiri

**Affiliations:** a Department of Dermatology, Brigham and Women’s Hospital, Boston, Massachusetts; b Harvard Medical School, Boston, Massachusetts; c University of Massachusetts Medical School, Worcester, Massachusetts; d Department of Pathology, Brigham and Women’s Hospital, Boston, Massachusetts

**Keywords:** drug-induced skin reactions, immune checkpoint inhibitors, pembrolizumab, Stevens-Johnson syndrome

## Introduction

Stevens-Johnson syndrome (SJS)/toxic epidermal necrolysis (TEN)—characterized by full-thickness epidermal cell death and extensive mucocutaneous reactions—is associated with high mortality and morbidity rates.^1^ SJS/TEN is typically attributable to medication exposure; common causative drugs include antibiotics, nonsteroidal anti-inflammatory drugs, anticonvulsants, barbiturates, and allopurinol.^1^

Although immune checkpoint inhibitors (ICIs)—including PD-1/PD-L1 and CTLA-4 inhibitors—have drastically improved therapy for a number of different cancers,^2^ ICIs also act on nonpathological cell types, resulting in immune-related adverse events. Cutaneous toxicities are among the most common immune-related adverse events from ICIs, with rates between 17% and 42% for PD-1 inhibitors as monotherapy.^2^^,^^3^ Higher grade and life-threatening dermatologic events, including SJS/TEN, occur in <1% of cases.^2^^,^^3^ We present a patient with an atypical case of pembrolizumab-induced SJS/TEN-like reaction, highlighting the importance of recognizing variable clinical and histopathologic presentations for accurate diagnosis, particularly in the setting of ICI therapy.

## Case

A 61-year-old woman with metastatic rectosigmoid adenocarcinoma presented with a 7-day history of nausea, vomiting, and intractable epigastric pain. She had received her second dose of pembrolizumab 8 days before admission. The first dose had been given 28 days prior, with no suspected side effects after the initial cycle. Her other medical history included type 2 diabetes and gastroesophageal reflux disease, treated with insulin and omeprazole, respectively.

Initial laboratory workup and abdominal computed tomography were unrevealing.

Physical examination was significant for white plaques on the superior aspect of the tongue concerning for thrush, which prompted oral fluconazole treatment. Two days after admission, the patient experienced periorbital swelling, a facial eruption of erythematous patches, and wheezing, which improved with diphenhydramine, albuterol, and methylprednisolone. Fluconazole was discontinued amid concerns of an adverse drug reaction.

**Fig 1:**
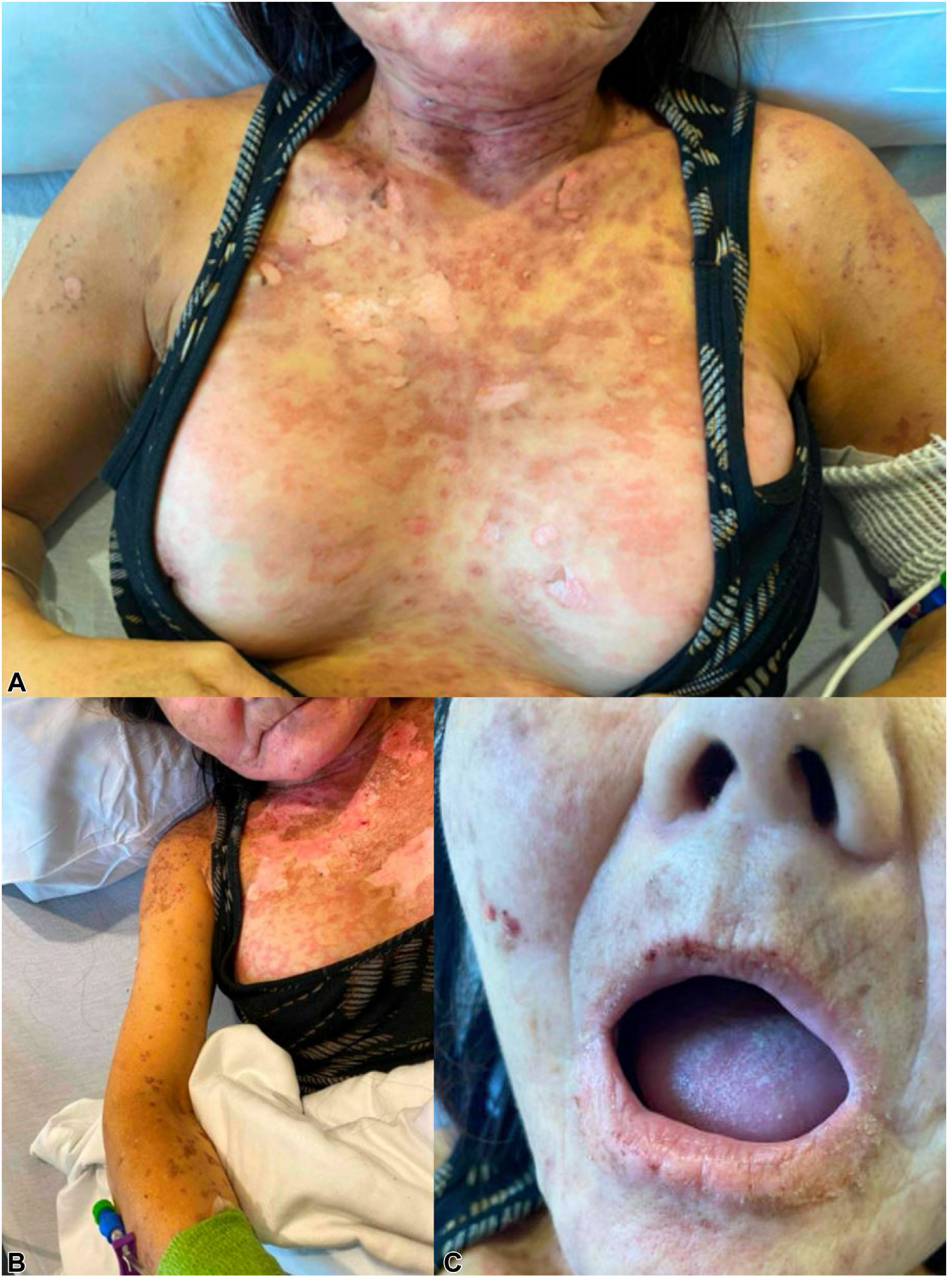
Gross dermatologic findings. **A,** Erythematous patches and plaques with scattered vesicles and bullae on chest. **B,** Scattered erosions overlying the chest that evolved to desquamating lesions with positive Nikolsky sign. **C,** Macerated erosions on bilateral oral commissures and buccal mucosa.

On the fifth day of admission, the patient’s periorbital edema worsened, and her cutaneous eruption expanded. She experienced pruritic, erythematous to dusky patches and plaques, studded with scattered vesicles and bullae over her abdomen, chest, back, neck, arms, and legs (Fig 1, *A*). Notably, she did not have skin tenderness or significant mucosal involvement.

**Fig 2:**
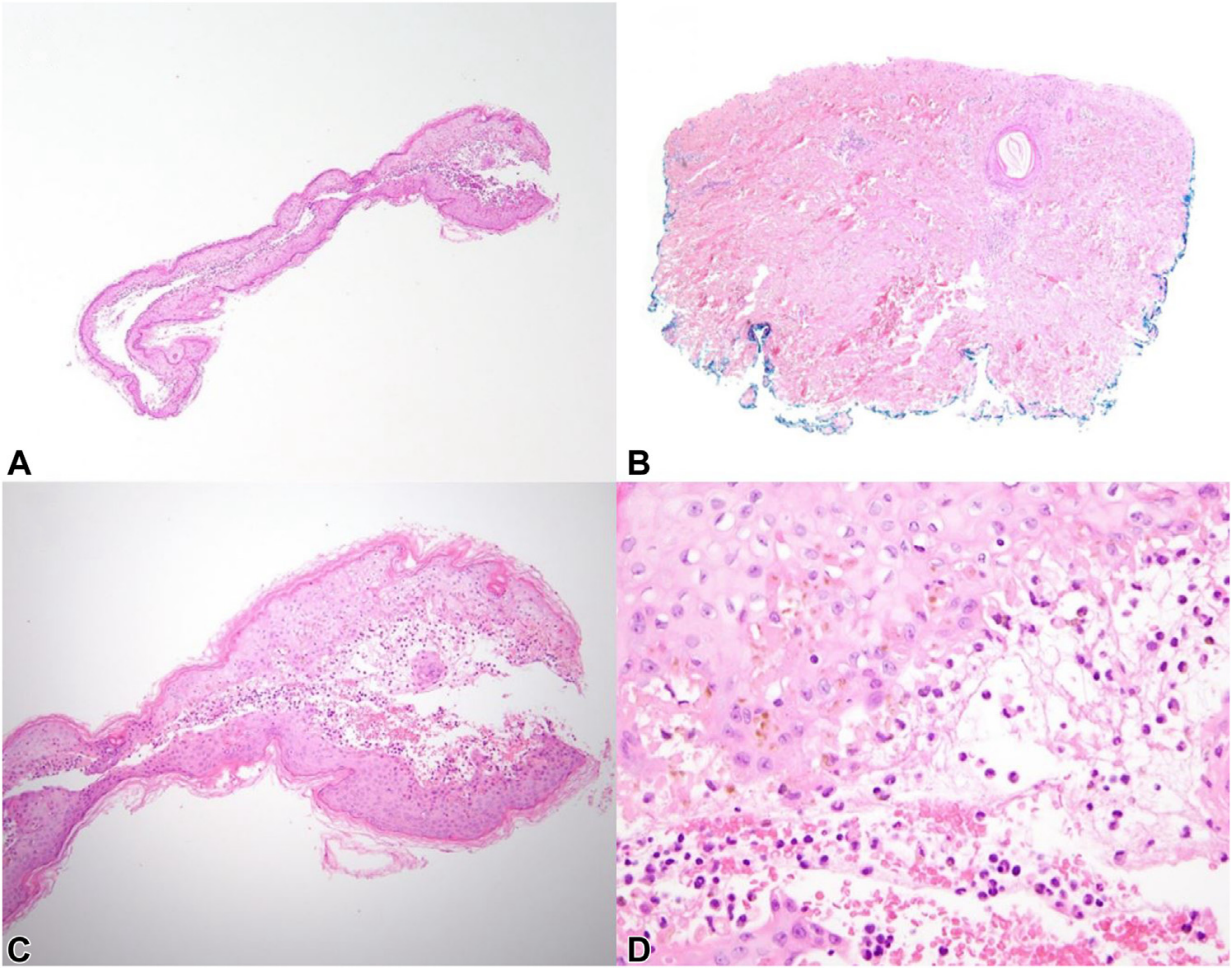
Histologic features of the abdominal punch biopsy. **A,** Detached fragment of epidermis secondary to subepidermal bulla formation. **B,** Denuded dermis underlying bulla. **C,D,** Prominent vacuolar interface dermatitis with marked dyskeratosis and mixed acute and chronic inflammation. (**A-D,** Hematoxylin-eosin stain; original magnifications: **A** and **B,** ×40; **C,** ×100; **D,** ×400.)

Initial histologic sections of a punch biopsy from the abdomen showed a detached fragment of epidermis secondary to subepidermal bulla formation (Fig 2, *A*) and denuded underlying dermis (Fig 2, *B*). Higher power evaluation revealed prominent interface dermatitis with marked dyskeratosis and mixed acute and chronic inflammation (Fig 2, *C*, *D*). Cumulatively, the findings consistent with an adverse cutaneous drug reaction but not necessarily specific for SJS/TEN. Anti-BP180 antibodies and anti-BP230 antibodies were not present, and IgE levels were normal.

On the sixth day of admission, the patient was subsequently started on oral prednisone without improvement; the eruption progressed with the formation of new painful flaccid bullae overlying the labia minora, extremities, and trunk that evolved to desquamating erosions with positive Nikolsky sign (Fig 1, *B*). This prompted the clinical diagnosis of pembrolizumab-induced SJS/TEN-like reaction.

One week later, oral ruxolitinib was initiated and intravenous steroids were tapered to prioritize antitumor effects of immunotherapy. Ondansetron and proton pump inhibitors were discontinued as potential associated triggers of her SJS/TEN. Subsequently, the eruption improved and no further new bullae emerged. She was transitioned to an oral prednisone taper and topical clobetasol 0.05% ointment. She was discharged 24 days after admission with healing skin and mucosal lesions.

Given the severity of the eruption, pembrolizumab was not reinitiated. Three months after hospitalization, surgical resection and restaging computed tomography showed no residual disease, demonstrating complete response of her rectosigmoid adenocarcinoma after 2 doses of pembrolizumab.

## Discussion

SJS and TEN are type IV cell-mediated delayed hypersensitivity reactions, typically presenting 1 to 3 weeks after exposure to an inciting drug.^1^ Skin lesions manifest as generalized patches that progress to large bullae with positive Nikolsky sign. The oral, urogenital, and periocular areas are often affected. The skin lesions are usually extremely painful and can lead to complications such as fluid loss, bleeding, hypothermia, and infection.^1^

Our patient’s clinical course was atypical. Although the initial presentation lacked extensive mucosal involvement and pain, the appearance of desquamating erosions and bullae were consistent with an erosive mucocutaneous disease or SJS/TEN. The initial histologic examination demonstrated a lack of full-thickness necrosis but was consistent with lichenoid infiltrate and dyskeratosis—pathologic findings that may be observed in early SJS.^4^ Cases such as ours describing ICI-induced SJS/TEN with a relative lack of mucosal involvement and similar histologic features may represent a unique clinical phenotype.^5^^,^^6^ Our patient had a Severity-of-Illness Score for Toxic Epidermal Necrolysis (SCORTEN; a prognostic metric to assess SJS/TEN severity and prognosis) score of 3, indicating a predicted mortality risk of >35.5%.^7^

Although our patient had been treated with multiple medications at the time of her cutaneous eruption, pembrolizumab was considered to be the most likely causative agent given the known prevalence of SJS/TEN-like eruptions secondary to ICIs.^4^^,^^5^ The algorithm of drug causality for epidermal necrolysis score, a validated algorithm for assessing drug causality in SJS/TEN, assigned a score of 4 to pembrolizumab, indicating “probable” likelihood of pembrolizumab being the causative agent over other medications.^8^ The median reported time to onset of SJS/TEN-like eruptions after ICI initiation is 6 to 6 weeks, consistent with our case.^4^^,^^6^

Although management of non–ICI-induced SJS/TEN is varied, current standards of care for ICI-induced SJS/TEN focus on the use of systemic corticosteroids, following National Comprehensive Cancer Network guidelines for high-grade dermatologic immune-related adverse events. However, steroid use in patients with extensive body surface area involvement, particularly >30%, remains controversial.^9^

Emerging evidence supports the use of intravenous immunoglobulin in cases refractory to systemic steroid monotherapy.^10^

For true SJS/TEN induced by ICIs, rechallenge with ICIs is contraindicated.^9^ For SJS/TEN-like eruptions, rechallenge can be considered in the setting of limited alternative therapeutic options. Although our patient had an atypical SJS/TEN-like eruption, pembrolizumab was permanently discontinued, and there was no recurrence. This case emphasizes the importance of considering SJS/TEN and SJS/TEN-like eruptions in patients receiving ICI therapy, despite atypical presentations, to provide timely and appropriate care.

## Conflicts of interest

None disclosed.
